# Efficacy and safety of vedolizumab for inflammatory bowel diseases: A systematic review and meta-analysis of randomized controlled trials

**DOI:** 10.1097/MD.0000000000030590

**Published:** 2022-10-07

**Authors:** Bo Qiu, Jia-Xu Liang, Cong Li

**Affiliations:** a International Doctoral School, University of Seville, Faculty of Medicine, Seville, Spain; b Department of Diagnostic Radiology, The Fifth Clinical Medical College of Henan University of Chinese Medicine (Zhengzhou People’s Hospital), Zhengzhou, China; c Department of Endocrinology of North District, The Second Affiliated Hospital of Zhengzhou University, Zhengzhou, China.

**Keywords:** Crohn disease, inflammatory bowel disease, ulcerative colitis, vedolizumab

## Abstract

Vedolizumab is a humanized monoclonal antibody that inhibits gut-selective α4β7 integrins on the surface of leukocytes, preventing their trafficking into the gastrointestinal tract, and ultimately achieves the effect of suppressing intestinal inflammation. This study aimed to evaluate the efficacy and safety of vedolizumab in the treatment of inflammatory bowel disease.

**Methods:**

After a systematic review of relevant studies, the pooled relative risk (RR) and 95% confidence intervals (CIs) were calculated to evaluate the effect. Heterogeneity was explored using sensitivity analysis, univariate meta-regression, and subgroup analysis. Potential publication bias was evaluated using Egger test and trim-and-fill method.

**Results:**

Nine randomized controlled trials involving 4268 participants were included in the meta-analysis. During induction therapy, vedolizumab was more effective than placebo in treating active ulcerative colitis and Crohn disease in terms of clinical response (RR = 1.55, 95%CI: 1.35–1.78), clinical remission (RR = 1.90, 95%CI: 1.50–2.41), and mucosal healing (RR = 1.53, 95%CI: 1.21–1.95). A superior effect in terms of durable Clinical or Crohn disease Activity Index-100 response (RR = 1.65, 95%CI: 1.20–2.26), clinical remission (RR = 1.92, 95%CI: 1.48–2.50), and glucocorticoid-free remission (RR = 2.22, 95%CI: 1.71–2.90) was found during maintenance treatment. Vedolizumab was not associated with any adverse events and was as safe as placebo in terms of the risk of serious adverse reactions.

**Conclusions:**

Vedolizumab may be safe and effective as an induction and maintenance therapy for the treatment of inflammatory bowel disease; however, further studies are needed to validate this conclusion.

## 1. Introduction

Inflammatory bowel disease (IBD) is a gastrointestinal disorder that includes ulcerative colitis (UC) and Crohn disease (CD), and is characterized by abdominal pain, chronic diarrhea, weight loss, and fatigue.^[1,2]^ IBD has become a global public health challenge in the past decade. In North America and Europe, >1.5 million and 2 million people suffer from the disease.^[3]^ Incidence rates have been increasing in newly industrialized countries in Africa, Asia, and South America since 1990. Unemployment, sick leave, and permanent work disability are more common in patients with IBD than in the general population.^[4]^ Moreover, there is a higher risk of adverse health outcomes, including multiple cancers, cardiovascular disease, adverse pregnancy outcomes, and other adverse events.^[5]^

Currently available medical treatments for IBD include immunosuppressants (e.g., azathioprine, mercaptopurine, and methotrexate), 5-aminosalicylates (5-ASAs), corticosteroids, and biological therapies such as tumor necrosis factor-alpha (TNF-α) antagonists.^[6–8]^ However, these medical therapies have limitations. 5-ASAs are only modestly effective^[9]^; a meta-analysis showed no statistically significant benefit in IBD patients receiving immunosuppressive therapy compared to placebo^[10]^; glucocorticoids can cause serious adverse effects and do not benefit from maintenance therapy^[11]^; TNF-α antagonists are effective but predispose patients to serious infection; and treatment failures may manifest as nonresponse or loss of response to these drugs over time.^[12,13]^ Therefore, new treatment strategies are required.

Vedolizumab is a humanized monoclonal antibody that inhibits the adhesion and migration of lymphocytes into the gastrointestinal tract by binding the alpha4beta7 (α4β7) integrin, which is a protein on the surface of lymphocytes targeted to the gastrointestinal tract.^[14,15]^ This disruption reduces inflammation of the gastrointestinal tract. Vedolizumab is indicated for the treatment of moderately to severely active UC and CD in patients with an inadequate response, loss of response, or intolerance to TNF-α inhibitors or other conventional therapies.^[16]^

Monitoring the efficacy and safety of vedolizumab is essential due to its relative newness and increasing number of patients being treated worldwide. The purpose of this study was to assess the efficacy and safety of vedolizumab induction and maintenance therapy in patients with IBD.

## 2. Materials and Methods

The Preferred Reporting Items for Systematic Reviews and Meta-Analyses (PRISMA) guidelines were adhered to as a methodological template for this review (Table S1, Supplemental Digital Content, http://links.lww.com/MD/xxx).^[17]^

### 2.1. Literature search strategy

Two investigators (B.Q. and J.X.L.) independently searched the MEDLINE (using PUBMED as the search engine), EMBASE, and Cochrane databases. Database were used to identify suitable studies published through April 2022. MeSH terms and keywords were used, and the search terms included: “inflammatory bowel diseases,” “ulcerative colitis,” “Crohn’s disease,” “vedolizumab,” “MLN0002,” “MLN02,” and “LDP-02.” The article type and additional filters did not restrict the search for published work. A manual search was conducted using the references listed in the original articles and the review articles retrieved. Two investigators collected the results separately.

#### 2.1.1. Inclusion criteria.

Randomized clinical trials (RCT);
Patients with active CD or UC;
Patients were treated with vedolizumab or placebo;

#### 2.1.2. Exclusion criteria.

Duplicate reports;

Studies conducted on animals;
Systematic reviews, meta-analyses, or nonrandomized controlled studies.

### 2.2. Data extraction

For each included study, all data elements uniformly reported across most studies were extracted by 2 reviewers (B.Q. and J.X.L.) and cross-verified by a third reviewer (C.L). When the same population was published in several journals, only the most informative articles or complete studies were retained to avoid duplication. The following information was extracted from each study: first author, year of publication, study design, sample size, diagnosis of enrolled patients, endpoint of the induction and maintenance phase, duration of follow-up, and adverse reactions.

### 2.3. Definition

Moderate-to-severely active UC was defined as a baseline full Mayo score of 6 to 12 with an endoscopic subscore ≥2.^[18]^ Clinical remission was defined as a total Mayo score of ≤2 and no individual subscore >1. Clinical response was defined as a reduction of ≥3 points and ≥30% from baseline in the full Mayo score. Mucosal healing was defined as an endoscopic subscore ≤1. Durable clinical response/remission was defined as clinical response/remission at the end of both the induction and maintenance phases. Corticosteroid-free remission was defined as clinical remission at the end of the maintenance phase without corticosteroid in patients who received concomitant corticosteroid therapy at week 0.

Moderate-to-severely active CD was defined as a baseline Crohn Disease Activity Index (CDAI) score of 220–450. Clinical response was defined as a ≥70-point decrease in CDAI from baseline. Clinical remission was defined as a CDAI score ≤150. The CDAI-100 response was defined as a ≥100 point reduction in the CDAI score from baseline.

### 2.4. Risk of bias assessment

The Cochrane risk-of-bias tool was used to assess the risk of bias in randomized trials.^[19]^ Two authors independently assessed each included article using this tool, and disagreements between the 2 authors were resolved by discussion with a third investigator.

### 2.5. Statistical analysis

Efficacy and safety were analyzed using dichotomous data, and the results were expressed as relative risk (RR) and 95% confidence intervals (CIs). The *I*^2^ statistic was used to measure the study heterogeneity, with *I*^2^ ≥ 50% indicating significant heterogeneity. If there was no heterogeneity, a Mantel–Haenszel fixed-effects model was used to calculate the pooled RRs, rather than a random-effects model. If heterogeneity was observed, univariate meta-regression or subgroup analysis was performed to explore different sources of heterogeneity. Sensitivity analyses were performed to determine whether there was an undue influence of a single study on the combined study results.^[20]^ We assessed potential publication bias using Egger test, with *P* > .05 indicating no publication bias. We also performed the Duval and Tweedie nonparametric “trim and fill” procedure to further assess the effect of publication bias in our meta-analysis. All statistical analyses were performed using Stata version 15 (Stata Corp, College Station, TX, USA) and RevMan 5.4 (The Cochrane Collaboration, Oxford, UK).

## 3. Results

### 3.1. Characteristics of the included studies

**Figure 1.**
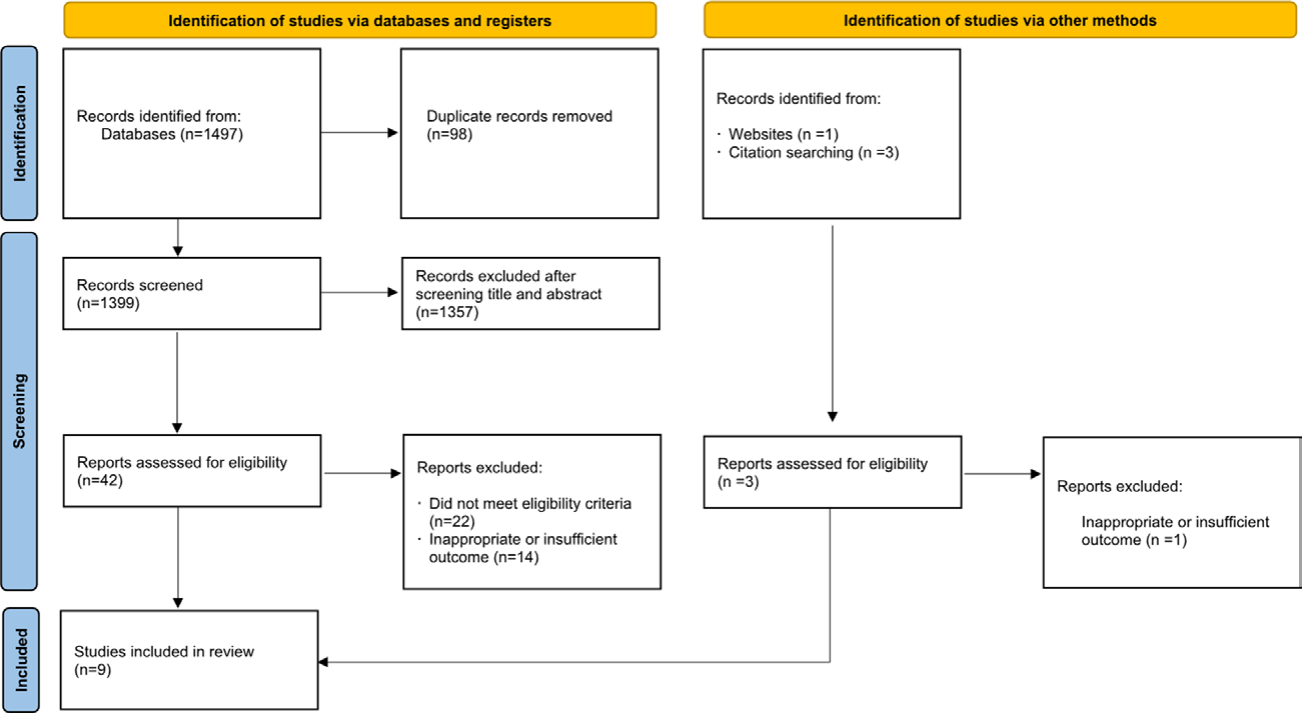
Study identification and selection flowchart.

Nine eligible RCTs were identified to evaluate the effectiveness and adverse events of vedolizumab in 4268 patients with active IBD.^[21–29]^ Figure 1 shows the identification and selection process of the study.

**Figure 2.**
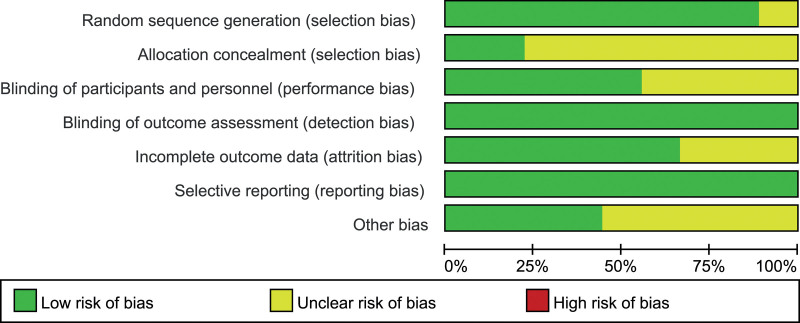
Evaluation of study quality.

In all studies conducted at multiple medical centers, the follow-up period ranged from 6 to 60 weeks. Four of the 9 studies included patients with active UC^[21,23,24,26]^ and 5 included patients with active CD.^[22,25,27–29]^ Three studies included patients who received open-label vedolizumab (cohort 2) in addition to randomized placebo-controlled trials (cohort 1).^[23–25]^ Eligible patients for inclusion in all studies needed to have evidence of active UC or CD and inadequate response, loss of response, or intolerance to at least 1 other treatment (corticosteroids, immunomodulators, or anti-TNF). Patients previously treated with vedolizumab, natalizumab, efalizumab, or rituximab were excluded from the respective included studies. The main characteristics of the included studies are summarized in Table 1. Before data analysis and synthesis, the Cochrane risk of bias tool was used to assess the quality of the studies, as shown in Figure 2.

**Table 1:** Study characteristics.

Author (yr)	Study design	Diseases	Sample size	Follow-up (wk)	Primary endpoint for the induction phase	Primary endpoint for the maintenance phase	The most common adverse events
Feagan 2005^[21]^	Double-blind	UC	Placebo: 63 VDZ: 118	6	Clinical remission		UC exacerbation, headache, nausea, frequent bowel movement, fatigue
Feagan 2008^[22]^	Double-blind	CD	Placebo: 58 VDZ: 127	8	Clinical response		CD exacerbation, headache, nausea, fatigue, nasopharyngitis
Feagan 2013^[23]^	Cohort 1: double-blind Cohort 2: open-label	UC	IP: 895 MP: 373	52	Clinical response	Clinical remission	Headache, UC exacerbation, nasopharyngitis, upper respiratory tract infection
Motoya 2019^[24]^	Cohort 1: double-blind Cohort 2: open-label	UC	IP: 292 MP: 83	60	Clinical response;	Clinical remission	Nasopharyngitis, and upper respiratory tract infection
Sandborn 2013^[25]^	Cohort 1: double-blind Cohort 2: open-label	CD	IP: 1115 MP: 461	52	Clinical response and clinical remission	Clinical remission	CD exacerbation, arthralgia, pyrexia, nasopharyngitis, headache, and nausea
Sandborn 2020^[26]^	IP: open-label MP: double-blind	UC	IP: 383 MP: 216	52		Clinical remission	Nasopharyngitis, anemia, and upper respiratory tract infection
Sands 2014^[27]^	Double-blind	CD	Placebo: 207 VDZ: 209	6	Clinical remission		Nausea, upper respiratory tract infection, arthralgia, abdominal pain
Vermeire 2022^[28]^	IP: open-label MP: double-blind	CD	IP: 644 MP: 410	52		Clinical remission	Nasopharyngitis, upper respiratory infections and gastrointestinal disorders
Watanabe 2020^[29]^	Double-blind	CD	IP: 157 MP: 42	54	CDAI-100 response	Clinical remission	Exacerbation of CD and upper respiratory tract infection

CD = Crohn disease, CDAI-100 = reduction in CD activity index score of ≥100 points from baseline, IP = induction phase, MP = maintenance phase, UC = ulcerative colitis, VDZ = vedolizumab.

### 3.2. Efficacy of vedolizumab for IBD

#### 3.2.1. Induction therapy.

##### 3.2.1.1. Clinical remission.

**Figure 3.**
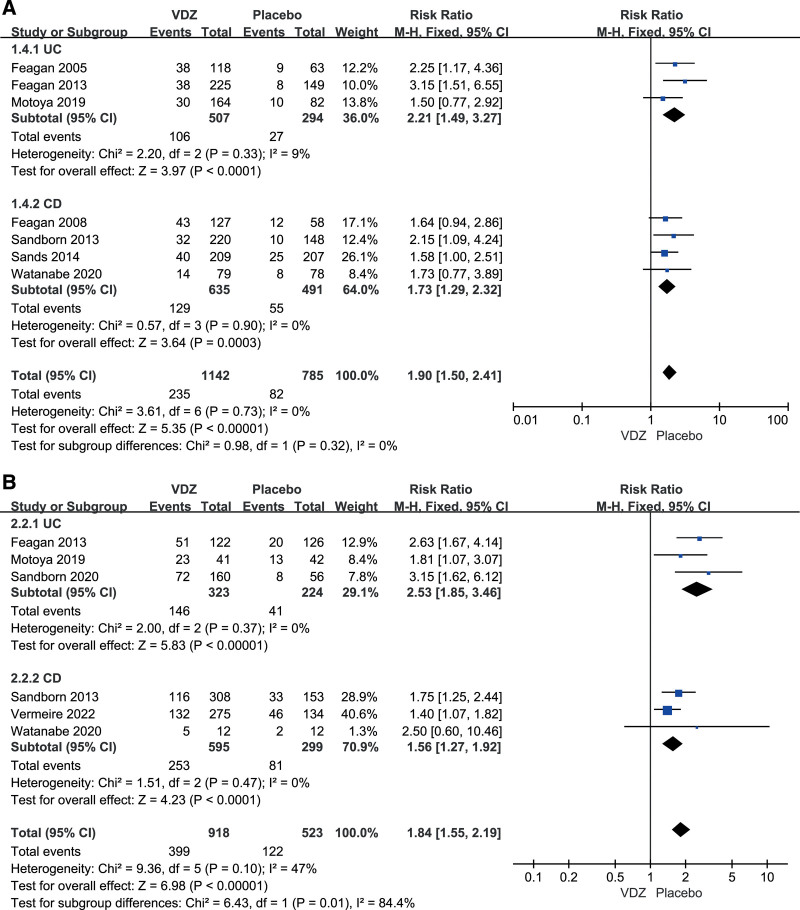
Subgroup analyses of clinical remission in (A) induction therapy and (B) in maintenance therapy.

Seven studies assessed the clinical remission after induction therapy.^[21–25,27,29]^ The results of the meta-analysis revealed that the clinical remission rate was significantly higher for patients who received vedolizumab than for those in the control group (RR = 1.90, 95%CI: 1.50–2.41). There was no heterogeneity between studies (*I*^2^ = 0%, *P* = .73). For UC patients as well as CD patients, a statistically significant difference between the vedolizumab and placebo groups was observed in our meta-analysis. Subgroup analyses were performed to assess the differential effects of vedolizumab in patients with UC and CD, and the results were similar to those of the overall analysis (Fig. 3A).

##### 3.2.1.2. Clinical or CDAI-100 response.

Three studies reported clinical responses in patients with UC^[21,23,24]^ and 4 reported CDAI-100 responses in patients with CD.^[22,25,27,29]^ The overall analysis showed that patients receiving vedolizumab had significantly higher clinical response rates than those receiving placebo (RR = 1.55, 95%CI: 1.35–1.78). There was no significant heterogeneity after pooling study data (*I*^2^ = 2%, *P* = .41). Similar results to the overall analysis were obtained for the UC and CD subgroups (Table 2).

**Table 2:** Efficacy and safety of vedolizumab versus placebo for IBD.

Category	Subgroup	RR (95% CI)	*P*	*I* ^2^
**Induction therapy**				
Clinical/CDAI-100 response	UC	1.62 (1.33–1.97)	<.05	43%
	CD	1.49 (1.23–1.80)	<.05	2%
Mucosal healing	UC	1.53 (1.21–1.95)	<.05	28%
	CD	NA		
**Maintenance therapy**				
Durable clinical remission	UC	2.12 (1.06–4.25)	<.05	0%
	CD	1.31 (0.86–2.01)	.21	0%
Clinical/CDAI-100 response	UC	2.15 (1.56–2.96)	<.05	0%
	CD	1.34 (1.13–1.59)	<.05	59%
Glucocorticoid-free remission	UC	2.44 (1.61–3.71)	<.05	0%
	CD	2.09 (1.48–2.94)	<.05	0%
**Safety**				
Adverse events	UC	1.03 (0.97–1.10)	.34	47%
	CD	1.00 (0.92–1.08)	.90	13%
Disease exacerbation	UC	0.90 (0.59–1.37)	.63	68%
	CD	0.64 (0.40–1.03)	.07	70%
Serious adverse events	UC	1.05 (0.78–1.42)	.08	0%
	CD	1.22 (0.97–1.52)	.08	57%
Serious infection	UC	0.68 (0.30–1.51)	.34	0%
	CD	1.12 (0.27–4.71)	.87	69%

CD = Crohn disease, CDAI = CD Activity Index, CI = confidence interval, IBD = inflammatory bowel disease, NA = not applicable, RR = relative risk, UC = ulcerative colitis.

##### 3.2.1.3. Mucosal healing.

Three studies reported the mucosal healing rate in induction therapy.^[21,23,24]^ A statistically significant difference was observed between the vedolizumab and placebo groups in the overall analysis (RR = 1.53, 95%CI: 1.21–1.95). No heterogeneity was observed among the studies (*I*^2^ = 28%, *P* = .25).

#### 3.2.2. Maintenance therapy.

##### 3.2.2.1. Clinical remission.

Six RCTs evaluated clinical remission in maintenance therapy.^[23–26,28,29]^ The overall analysis showed significantly higher clinical remission rates in patients receiving vedolizumab compared to those in the control group (RR = 1.92, 95%CI: 1.48–2.50). Heterogeneity was observed among these studies (*I*^2^ = 47%, *P* = .10). The results in the UC and CD subgroups were similar to those of the overall analysis (Fig. 3B).

Four studies reported durable clinical remission with maintenance therapy.^[24–26,29]^ A statistically significant difference was found between the vedolizumab and placebo groups in the overall analysis (RR = 1.52, 95%CI: 1.06–2.19, *I*^2^ = 0%). However, in the sub-analysis, there was no statistically significant difference in CD patients (RR = 1.31, 95%CI: 0.86–2.01) (Table 2).

##### 3.2.2.2. Durable clinical or CDAI-100 response.

Two studies reported durable clinical responses in patients with active UC,^[24,26]^ and 3 studies reported the durable CDAI-100 response in patients with active CD.^[25,28,29]^ A significant difference was found between the vedolizumab and placebo groups in the overall analysis (RR = 1.65, 95%CI: 1.20–2.26). However, there was heterogeneity between these studies (*I*^2^ = 68%, *P* = .01), therefore, a random-effects model was used. Our meta-analysis revealed statistically significant differences between the vedolizumab and placebo groups in UC patients as well as in CD patients (Table 2).

##### 3.2.2.3. Glucocorticoid-free remission.

Six studies evaluated glucocorticoid-free remission during maintenance therapy.^[23–26,28,29]^ The overall analysis showed significantly higher glucocorticoid-free remission rates in the vedolizumab group than in the control group (RR = 2.22, 95%CI: 1.71–2.90). There was no heterogeneity among the studies (*I*^2^ = 0%, *P* = .92). The results of the UC and CD subgroups were similar to those of the overall analysis (Table 2).

### 3.3. Clinical remission in subgroups based on prior TNF antagonist use status

**Figure 4.**
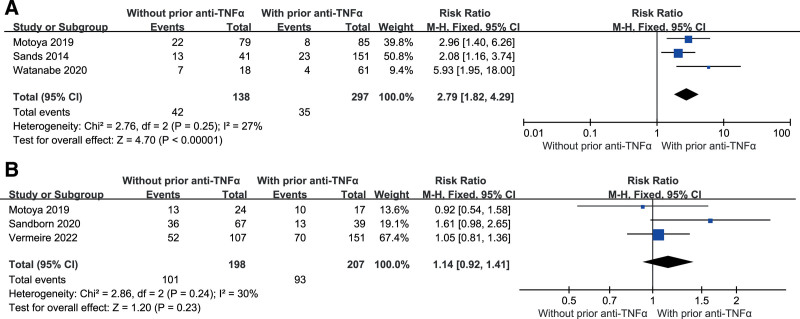
Clinical remission in subgroups based on prior TNF antagonist use status in (A) induction therapy and (B) in maintenance therapy. TNF-α = tumor necrosis factor-alpha.

During the induction phase with vedolizumab, clinical remission rates were higher in anti-TNF-naive patients than in patients with prior use of anti-TNF (RR = 2.79, 95%CI: 1.82–4.29, *I*^2^ = 27%). However, there was no significant difference between the 2 groups during the maintenance phase (RR = 1.14, 95%CI: 0.92–1.41, *I*^2^ = 30%) (Fig. 4).

### 3.4. Safety of vedolizumab for IBD

#### 3.4.1. Adverse events.

Seven studies reported adverse events during the follow-up period.^[22–24,26–29]^ Adverse events included disease exacerbation, nasopharyngitis, upper respiratory tract infection, nausea, vomiting, abdominal pain, headache, arthralgia, and fatigue. No statistically significant difference between the vedolizumab and placebo groups (RR = 1.01, 95%CI: 0.96–1.07, *I*^2^ = 25%).

Seven RCTs reported exacerbation events of UC or CD.^[21–23,25,27–29]^ Based on the overall analysis, there was no statistically significant difference between these 2 groups (RR = 0.77, 95%CI: 0.57–1.03, *I*^2^ = 64%) (Table 2).

#### 3.4.2. Serious adverse events.

All included studies reported serious adverse events during the follow-up.^[21–29]^ In the overall analysis, there were no statistically significant differences between the vedolizumab and placebo groups (RR = 1.16, 95%CI: 0.97–1.39, *I*^2^ = 32%).

Serious infections were reported in 6 studies.^[21,23–25,27,28]^ The difference between the vedolizumab and placebo groups was not statistically significant (RR = 0.91, 95%CI: 0.40–2.08, *I*^2^ = 51%) (Table 2).

### 3.5. Heterogeneity analysis

Significant heterogeneity was observed in “durable clinical or CDAI-100 response in maintenance therapy (*I*^2^ = 68%),” “UC or CD exacerbation (*I*^2^ = 64%),” and “serious infection events (*I*^2^ = 51%).” Hence, we conducted a meta-regression analysis to examine the sources of potential heterogeneity based on the following predefined characteristics: diagnosis (UC vs CD) and study design (open-label vs non-open-label). The results showed that “patient diagnosis,” and “study design” were not factors contributing to heterogeneity (Table 3).

**Table 3:** Univariate meta-regression analysis.

Category	*P* value	
	Diagnosis	Study design
DC or CDAI-100 response in MP	.85	.54
Disease exacerbation	.67	.88
Serious infection	.94	.45

DC = durable clinical, MP = maintenance phase.

Sensitivity analysis was performed by removing 1 study and recalculating the pooled estimates for the remaining studies, which showed that the pooled results were not significantly affected by the individual studies.

### 3.6. Publication bias

All *P* values of the Egger statistical tests were >0.05. Although Egger test was not statistically significant, visual inspection of the funnel plot revealed asymmetry, which raised the possibility of publication bias. Hence, we performed a sensitivity analysis using the trim-and-fill method.^[30]^ The difference between the original and corrected effect size estimates was not significant, suggesting that publication bias did not affect the results (Table 4).

**Table 4:** The overall effect sizes before/after applying the trim-and-fill methods.

Category	Result of Egger test	Imputed missing studies	Before trim-and-fill methods	After trim-and-fill methods
Clinical remission in IP	*P* = .33	1	1.97 (1.57–2.38)	2.05 (1.66–2.44)
CDAI-100/clinical response in IP	*P* = .93	0	1.57 (1.43–1.70)	1.57 (1.43–1.70)
Mucosal healing in IP	*P* = .81	1	1.53 (1.29–1.77)	1.46 (1.23–1.69)
Clinical remission in MP	*P* = .20	3	2.14 (1.61–2.67)	1.62 (1.06–2.19)
DC remission in MP	*P* = .37	1	1.69 (1.05–2.33)	1.94 (1.24–2.64)
CDAI-100/DC response in MP	*P* = .10	2	1.47 (1.32–1.62)	1.30 (1.16–1.45)
GFR in MP	*P* = .29	1	2.32 (1.93–2.72)	2.29 (1.92–2.67)
Adverse events	*P* = .77	1	1.04 (0.99–1.08)	1.04 (1.00–1.09)
Serious adverse events	*P* = .26	4	1.20 (1.02–1.38)	1.34 (1.18–1.50)
Serious infection	*P* = .87	1	1.49 (0.45–2.53)	1.20 (0.08–2.31)
Disease exacerbation	*P* = .17	2	0.87 (0.72–1.03)	0.90 (0.74–1.07)

CDAI = CD Activity Index, DC = durable clinical, GFR = glucocorticoid-free remission, IP = induction phase, MP = maintenance phase.

## 4. Discussion

Inflammatory bowel disease (IBD) is a multifactorial chronic disease that may be associated with lifestyle, surgery, living environment, and inappropriate inflammatory responses to intestinal microbes in genetically susceptible individuals.^[31,32]^ Patients with IBD are at a higher risk of adverse health outcomes.^[5]^ Although conventional treatments to induce remission of moderately to severely active UC and CD include sodium 5-ASAs, corticosteroids, and immunomodulators, treatment effects have been unsatisfactory owing to a few limitations or serious adverse events.^[33–35]^ Vedolizumab specifically antagonizes the intestinal α4β7 integrin heterodimer, which prevents lymphocytes from migrating and homing from the blood to intestinal tissues, ultimately inhibiting intestinal inflammation.^[36]^ Vedolizumab is considered a first-line biological treatment for UC and CD.^[37]^

In this meta-analysis, we identified 9 randomized, placebo-controlled clinical trials that evaluated vedolizumab in the treatment of CD or UC. Our study demonstrated that vedolizumab improved clinical remission and clinical response in patients with active UC, as well as clinical remission and CDAI-100 response in patients with active CD in both induction and maintenance therapy, which indicated that vedolizumab enhances the relief of patient-perceived symptoms. However, it is important to consider that vedolizumab had no effect on durable clinical remission in patients with CD. As reported by Vermeire et al,^[28]^ clinical remission at week 52 was similar between vedolizumab and placebo in the anti-TNF-naive population (*P* = .59). Watanabe et al^[29]^ also showed no significant difference between the vedolizumab and placebo groups in terms of durable clinical remission (*P* = .65). In addition, the vedolizumab group exceeded the placebo group at all endpoints, regardless of prior anti-TNF-α use, except for durable remission in patients without prior anti-TNF-α use. Thus, a possible confounding factor is whether the patients had a previous exposure to anti-TNF therapy. Our subgroup analysis based on the status of prior anti-TNF use, demonstrated a higher rate of clinical remission in TNF antagonist-naive patients during induction therapy. A multicenter cohort study by Amiot et al^[38]^ evaluated the efficacy of vedolizumab in IBD patients with prior TNF antagonist failure, resulting in clinical remission rates of 36% and 39% in the CD and UC groups, respectively, after 14 weeks of induction therapy. Kopylov et al^[39]^ evaluated the efficacy of vedolizumab in patients without previous anti-TNF-α use. The results showed that vedolizumab was as effective in IBD patients without previous anti-TNF-α use and had significantly improved efficacy in patients with previous anti-TNF-α failure. Mucosal healing and glucocorticoid-free remission were also significantly higher in the vedolizumab group. Mucosal healing is an important IBD treatment goal that is associated with sustained clinical remission, glucocorticoid-free remission, and reduced incidence of hospitalization and surgery.^[40]^ In our systematic review, more than half of patients with UC achieved mucosal healing, with a significant benefit compared with placebo (55.1% vs 22.8%).

The most frequently observed adverse effects in patients treated with vedolizumab included nasopharyngitis, headache, nausea, arthralgias, pyrexia, fatigue, upper respiratory tract infections, cough, and abdominal pain.^[41]^ Wang et al^[42]^ showed a higher incidence of serious adverse events with vedolizumab in patients with CD (21.7% vs 14.3%) than with placebo which is similar to the results of Sandborn et al^[25]^ (24.4% vs 15.3%). In this study, the incidence of serious adverse events was higher (17.3% vs 11.7%) during induction therapy, but was comparable between the 2 treatment groups during maintenance therapy (11.1% vs 12.3%). In patients with CD, the incidence of serious adverse events was higher compared to patients with UC (19.2% vs 12.4%). This may be because CD is a systemic disease with multi-organ involvement, as it may affect any part of the gastrointestinal tract.^[43]^ Our results showed that vedolizumab was not associated with a greater incidence of adverse events and was as safe as the placebo in terms of the risk of serious adverse reactions, which also included the risk of serious infections. Although the risk analysis of drug-related adverse events in the treatment of IBD has not been well studied, the use of vedolizumab may be a better option than immunosuppressants, corticosteroids, or TNF-α antagonists in treating patients at higher risk for serious infections, such as the elderly or patients with chronic lung disease (e.g., chronic obstructive pulmonary disease).^[44]^ Therefore, a prospective evaluation of this possibility is required.

Sandborn et al^[26]^ showed that a subcutaneous (SC) formulation of vedolizumab (108 mg administered every 2 weeks) is effective and safe as maintenance therapy for patients with moderately to severely active UC. Furthermore, the European Commission has approved a subcutaneous formulation of vedolizumab based on the Sandborn pivotal Phase 3 VISIBLE trial. The subcutaneous formulation of vedolizumab will provide an additional option for patients to maintain a clinical response to vedolizumab. In 2 pilot studies on maintenance therapy, patients who responded to vedolizumab at week 6 were randomly assigned to receive placebo or vedolizumab every 8 or 4 weeks up to 52 weeks.^[23,25]^ Patients treated with vedolizumab every 8 and every 4 weeks differed significantly from those treated with placebo who were in clinical remission at week 52. However, there was no significant difference for the 2 groups treated with vedolizumab.

Although the included studies were multicenter randomized controlled studies, there were some limitations. First, it was not the purpose of the present study to identify the duration of greatest effect of vedolizumab as induction therapy. Extension of induction therapy beyond 6 weeks may result in greater efficacy. Second, the efficacy of the maintenance therapy was not designed to be statistically evaluated in some studies. The relative efficacy and safety of vedolizumab compared with other IBD therapies, particularly the TNF-a antagonists, adalimumab, infliximab, and golimumab, should also be evaluated in future studies. It is difficult to make recommendations on the initial choice of biologic therapy for biologic-naive patients in the absence of direct comparisons. Additionally, the number of included RCTs was small, and most of the included RCTs did not report specific details of drug-related serious adverse events. Moreover, including only English-language studies might lead to better results because studies with positive results are more likely to be accepted by an international journal.

In conclusion, the results of this meta-analysis showed that vedolizumab was significantly more effective than placebo as an induction and maintenance treatment for IBD. Importantly, serious adverse events were not more common in vedolizumab-treated patients than in the control patients. However, the number of studies included for the analysis was significantly smaller, necessitating a reanalysis when more data became available.

## Author contributions

Study concept and design: BQ, JXL, CL. Data collections: BQ, JXL. Data analysis and interpretation: BQ, JXL. Drafting of the manuscript: BQ, JXL. Critical revision of the manuscript for important intellectual content: CL. All authors read and approved the final manuscript. Bo Qiu and Jiaxu Liang contributed equally to this work.

Conceptualization: Bo Qiu.

Data curation: Jia-Xu Liang.

Formal analysis: Bo Qiu.

Investigation: Bo Qiu.

Methodology: Bo Qiu, Jia-Xu Liang.

Supervision: Bo Qiu, Cong Li, Jia-Xu Liang.

Validation: Bo Qiu, Cong Li, Jia-Xu Liang.

Visualization: Bo Qiu, Cong Li, Jia-Xu Liang.

Writing – original draft: Bo Qiu.

Writing – review & editing: Bo Qiu, Jia-Xu Liang.
